# Effect of calcitriol supplementation and tail suspension on serum biomarkers of bone formation in rats

**DOI:** 10.1186/s40200-015-0142-5

**Published:** 2015-03-19

**Authors:** Seyed Jafar Hashemian, Mojtaba Rismanchi, Ensiyeh Nasli Esfahani, Amir Khoshvaghti, Farideh Razi

**Affiliations:** Diabetes Research Center, Endocrinology and Metabolism Clinical Science Institute, Tehran University of Medical Sciences, Tehran, Iran; Department of Neurology, Shiraz University of Medical Sciences, Shiraz, Iran; Faculty of Aerospace and Sub-Aquatic Medicine, AJA University of Medical Sciences, Tehran, Iran

**Keywords:** Tail suspension model, Vitamin D, Calcitriol, Osteocalcin (OC), Alkaline phosphatase (ALP)

## Abstract

**Background:**

Calcitriol is documented to cause significant increase in bone mass densitometry counteracting osteoporosis. Promising results of calcitriol supplementation in studies aiming space flight induced osteoporosis is little and the effect of this hormone on biomarkers of bone metabolism is not examined yet in space flight models of osteoporosis in rats.

**Methods:**

This was an interventional animal study being performed in a 1-month period. We included 21 Sprague Dawley strain rats (>200 gr, >6 week) who were randomly assigned to receive daily supplementation of oral 0.03μgr calcitriol and to be submitted to tail suspension model. Rats were followed for 1 month and were tested for serum osteocalcin (OC), alkaline phosphatase (ALP) and serum calcium at the beginning and the end of the study period. The results were analyzed and compared between groups.

**Results:**

Although serum levels of osteocalcin and alkaline phosphatase biomarkers and total serum calcium were not significantly different within and between study groups, their levels were increased in tail suspension model compared to control group. The levels of these biomarkers were lower in those who were submitted to tail suspension model and received calcitriol supplementation compared to those who were only submitted to tail suspension (*60.14 ± 11.73 ng/mL vs. 58.29 ± 2.69 ng/mL; p = 0.696 for osteocalcin and 381.86 ± 99.16 mU/mL vs.* 362.57 ± 27.41 *ng/mL; p = 0.635 for alkaline phosphatase*).

**Conclusion:**

Supplementation of daily diet with calcitriol in rats under weightlessness conditions may results in lower values for bone metabolic biomarkers of alkaline phosphatase and osteocalcin and serum calcium. This pattern of change in biomarkers of bone formation, may point to the capacity of calcitriol supplementation in preventing cellular process of osteoporosis. Thus calcitriol supplementation could be an available, economic and effective strategy for preventing bone metabolic changes related to weightlessness commonly encountered in space flight. The outcome of this study needs to be further studied in future trying to find more definite results.

## Introduction

During space flight, calcium metabolism is directed toward resorption from bone and excretion into urine, all of which leads to bone loss and renal stone formation [1]. Factors affecting calcium metabolism in these situations include low dietary intake of calcium, low lighting, increased CO2 levels in the space station environment and finally the most important factor is skeletal unloading [2]. Bone loss and its consequential osteoporosis, is more exaggerated in the lower extremities of out of space voyagers, which point to the unloading as the most important factor of bone loss [3]. Follow up studies of long duration space flights including early studies of Mir and Skylab missions and recent study of NASA bone summit have shown that bone loss could become up to 15% that was followed by only 6% of bone recovery after 1 year on earth [2,4,5]. These observations lead researchers to address the issue of the space exploration limitation due to osteoporosis [6].

Pharmacological interventions to prevent the rate of bone loss in situations of weightlessness are the focus of interest in the literature [7,8]. The latest NASA recommendation for preventing the rate of bone loss and hence osteoporosis also included the use of pharmacological interventions in the studies designed for such purposes [5]. Several pharmacologic interventions have been tested in order to prevent or reverse disuse osteopenia. These include the use of antiabsorptive agents like alendronate and anabolic drugs like estrogen [7]. Supplementation of daily diet with vitamins and elements acting on bone including vitamin K and D is another field of interest in preventing disuse osteoporosis [7,8]. These studies demonstrated that vitamin D as contrary to vitamin K does not show promising results in studies involving astronauts [8].

One of the disadvantages for designing trial studies concerning the improvement in bone mineral density is the rare and expensive settings of trial in out of space or scarce situations mimicking this condition. Therefore, the use of animal models for performing studies mimicking weightlessness is becoming more widespread among researchers [9,10] in recent years, the use of unloaded rodents as a model of weightlessness that is well known in the literature as hindlimb suspension model is becoming the model of choice for simulating space flight [11,12]. The influence of different measures for reducing the detrimental effects of weightlessness on animal bone has been tested by this model till now. These include the study of bisphosphonates, testestrone and vitamin K on bone markers of tail suspended rats [13-15]. The effect of vitamin D on bone metabolism and bone turn over markers of out of space voyagers is not demonstrated yet; therefore in this article the effect of vitamin D on bone forming biomarkers of hindlimb suspended rats is studied.

## Material and methods

### Study animals and grouping

Dawley strain male rats were raised to 6 weeks of age weighing 220 ± 20 grams at Pastour institute laboratory and breeding center (Tehran, Iran). At 7 week of age 19 rats were transferred to animal housing and laboratory affiliated to Iran university of medical sciences (Tehan, Iran). All rats were housed for 1 week before tail suspension experiment in collective cages with standard conditions of animal care including that of temperature (23 ± 2°C) and free access to food and water. They were randomly assigned into three groups: baseline control group (n = 7, no intervention, no tail suspension) which was observed and two subgroups (each n = 7), both were submitted to tail suspension. The experimental study was approved by the Ethics Committee on Animal Research of AJA University of Medical Sciences, with approval code of 691180 in 31 January 2012.

### Study protocol and intervention

One group (n = 5) was housed in conventional CP-3 cages receiving general laboratory care for 1 month during the experiment. Two other groups (n = 7, each) were submitted to modified metabolic cages under hindlimb unloaded condition through tail suspension for 1 month. The suspension method was performed Morey-Holton and Wronski [11,16]. In this technique the rats were anesthetized (xylazine 30 mg/kg body weight and ketamine 30 mg/kg body weight) and traction tape was applied at the base of the tail. The prepared rats were then attached to the top part of metabolic cages equipped with suspension device. This technique of unloading was previously used successfully in rats and it has become the method of choice for space flight simulation [11]. It is also shown that animals gain weight during tail suspension indicating less stressful nature of the technique in comparison to other methods like back harness [16]. During the suspension period the rats had free access to food and water and they were kept in standard conditions of laboratory care with 12-h light/dark cycle.

One suspended subgroup (n = 7) was given 4 IU/Kg calcitriol (1,25 2OH vitamin D3) suspension in daily water. Calcitriol (an oil soluble drug) was changed into water suspension drug form by I-Safshekan D-Pharm (Baabak pharmacy, Shiraz, Iran).

### Sampling and measurements

The rats were sampled for their blood in the beginning and the end of the experiment. Sera from blood samples were collected and placed in minus 80°C freezer. All samples are tested at the end of the experiment for serum calcium and markers of bone formation including ALP and OC in the Endocrinology and Metabolism Research Institute, Tehran University of Medical Sciences, Tehran, Iran. OC was measured using commercially available rat specific Osteocalcin® laboratory Eliza kit purchased from Hangzhou Eastbiopharm Co., Ltd. (Hangzhou, China) and the tests were run as recommended by the manufacturer. Calcium and ALP were measured by spectrometry and enzymatic techniques respectively in the Endocrinology and Metabolism Research Institute affiliated to Tehran University of Medical Sciences, Tehran, Iran.

### Statistical analysis

In order to have 90% power to detect significant differences between changes in biomarkers of bone metabolism, 7 animals were required in each intervention subgroups and 5 rats in the control group. The Statistical Package for Social Science, SPSS for Windows, version 16.0 (SPSS, Chicago, IL) was used for data analysis. Paired t-tests were used to compare results within groups; independent t-tests were used to compare results between the groups. Data are reported as means ± SD. A two-sided p-value less than 0.05 was considered statistically significant.

## Results

A total number of 21 rats were included in the study that all finished the study period. Three study groups, out of which two groups were submitted to hindlimb suspension through tail suspension and one control group was selected from the same strain and under the same conditions including that of food and water except for the calcitriol supplementation of one suspended group.

Table 1 summarizes the main outcomes of the study. There was no significant difference within and between all study groups regarding the baseline measurements for OC and ALP biomarkers (Tables 1 and 2). Those who received calcitriol and were submitted to hindlimb suspension during the study had lower values for OC and ALP at the end of the study compared to the only suspended group (*53.43 ± 7.27 ng/mL vs. 60.14 ± 11.73 ng/mL; p = 0.227 for osteocalcin, 370.71 ± 84.02 mU/mL vs. 381.86 ± 99.16 mU/mL; p = 0.824 for alkaline phosphatase*) and higher values regarding ALP compared to the control group (370.71 ± 84.02 *mU/mL vs.* 362.57 ± 27.41 *mU/mL*; p = 0.814) and lower values regarding OC compared to control group (*53.43 ± 7.27 ng/mL vs. 58.29 ± 2.69 ng/mL; p = 0.138*) though the differences were not significant. The drug supplemented group also had lower serum calcium (9.61 ± 0.54 *vs.* 9.75 ± 0.42; p = 0.596) compared to the only suspended group, though the differences were not significant.

**Table 1:** **Test results of 20 rats who received tail suspension and calcitriol, tail suspension or none (Independent sample** ***T*** **-test)**

	**Osteocalcin (ng/mL)**	**ALP (mU/mL)**	**Serum calcium (mg/dL)**
Suspended group and calcitriol supplementation (n = 7)	53.43 ± 7.27	370.71 ± 84.02	9.61 ± 0.54
Only suspended group (n = 7)	60.14 ± 11.73	381.86 ± 99.16	9.75 ± 0.42
Control group (n = 7)	58.29 ± 2.69	362.57 ± 27.41	9.78 ± 0.23

Values are presented as mean ± SE. The data were not significant between groups.

**Table 2:** **Study measurements at the beginning and the end of the experiment and their comparison (paired** ***T*** **-test)**

	**Beginning of the study**	**End of the study**	**P- value**
Suspended group and calcitriol supplementation (n = 7)			
Osteocalcin (ng/mL)	59.71 ± 1.79	53.43 ± 7.27	0.05
ALP (mU/mL)	357.86 ± 21.76	370.71 ± 84.02	0.68
Serum calcium (mg/dL)	9.77 ± 0.22	9.61 ± 0.54	0.55
Only suspended group (n = 7)			
Osteocalcin (ng/mL)	57.71 ± 2.69	60.14 ± 11.73	0.66
ALP (mU/mL)	344.71 ± 12.459	381.86 ± 99.16	0.31
Serum calcium (mg/dL)	9.82 ± 0.19	9.75 ± 0.42	0.59
Control group (n = 7)			
Osteocalcin (ng/mL)	58.14 ± 2.61	58.29 ± 2.69	0.93
ALP (mU/mL)	357.86 ± 26.36	362.57 ± 27.41	0.53
Serum calcium (mg/dL)	9.8 ± 0.23	9.78 ± 0.23	0.85

Values are presented as mean ± SE.

## Discussion

**Figure 1:**
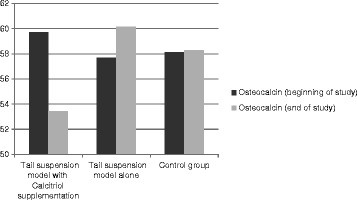
**Comparison of test value for osteocalcin biomarker at the beginning and the end of study within and between groups.** (Data were not significant).

**Figure 2:**
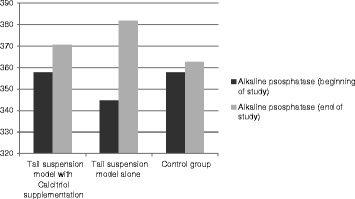
**Comparison of test value for ALP biomarker at the beginning and the end of study within and between groups.** (Data were not significant).

**Figure 3:**
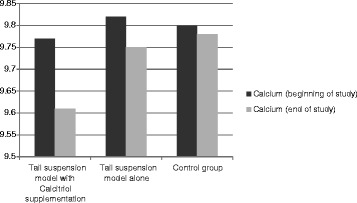
**Comparison of test value for serum calcium at the beginning and the end of study within and between groups.** (Data were not significant).

During space flight physiologic changes happen in the bone metabolism in the body of astronauts in response to microgravity. These include decrease in calcitriol levels along with increase in serum calcium content pointing to the release of calcium from bone tissue [8,17]. Furthermore during tail suspension model in animals, it is documented that serum levels of OC and ALP also increase in response to unloading [18]. New interventions are now underway to prevent osteoporosis during weightlessness by preventing this process. In this article we have tested the effect of activated vitamin D supplementation on serum calcium and biomarkers of bone formation. Our data though did not become statistically significant are in favor of lower serum values for OC, ALP and calcium in the tail suspended group receiving calcitriol supplementation compared to the tail suspended group alone (Figures 1, 2 and 3). The study was also in favor of the increased values for OC, ALP and calcium in tail suspended group alone, all point to increased bone turnover in this group leading to higher serum calcium values, which is consistent with the previously reported results for tail suspension model in mice [18] (Figures 1, 2 and 3).

Bone metabolism is the result of two opposite mechanisms involved in bone turn over. These include bone formation and resorption happening continuously as the tissue evolves [19]. Several markers of bone turnover each assessing specific physiolocal properties of this process have been introduced till now [20]. These markers have been used for assessing different pathologies related to bone tissue like metabolic bone disorders, complications of different metastatic diseases involving bone and risk of fractures and even diseases not involving bone and skeletal system [21-23]. Successful relationships are also established between bone markers of turnover and bone mass densitometry and fracture risk [24]. One important aspect of using bone turnover markers is their capacity to detect early bone responses to treatment regimens [20] while on a molecular point of view, these markers provide researchers with novel opportunities to evaluate bone cellular responses to new interventions [25].

An increased serum level of ALP, a bone formation marker, is correlated with low BMD scores which points to the enhanced osteoblastic activity in coping with bone loss [26]. This finding has lead to the reinforcement of endocrine society to conduct studies for finding interventions capable of reducing this marker [26]. The administration of vitamin D in vitamin D deficient patients is shown to reduce this biomarker [27,28]. On the other hand increase in ALP is shown to correlate with the improvement in bone mass densitometry during treatment with parathyroid hormone analogues in osteoporosis [29]. These findings point to ALP, to be a marker of bone formation and surrogate marker of bone loss, all together indicating the need for couscous and proper interpretation of the change in this biomarker.

Although vitamin D supplementation is documented to cause significant increase in bone mass densitometry and decrease in the risk of bone fracture [30,31], the effect of this vitamin on OC that is another biomarker of bone formation is still unclear. Regarding this marker, it is shown that OC is increased after treatment with vitamin D, but this change in OC level was repeatedly reported to be not significant in the literature [32,33]. Furthermore it is demonstrated that unlike vitamin K, vitamin D does not change undercarboxylated OC ratio with OC [32]. All of these documents point to the equivocal response of bone tissue to the administration of vitamin D supplement.

Weightlessness situations cause decrease in PTH level which in turn causes decrease in calcitriol level with the net result for serum calcium content to become dependent on skeletal calcium resorpsion [34]. Although a rare presentation, hypercalcemia mediated by calcitriol is reported in specific patients like sarcoidosis and lymphoma [35,36], here we have shown decrease in serum calcium content after administration of calcitiol along with suppression of bone metabolic markers including ALP ad OC. This shows hypometabolic state of bone metabolism and decrease in serum calcium content similar to the effect of steroid on bone metabolism [37]. Altogether these findings indicate antiresorptive effect of calcitriol on bone metabolism. The inability of calcitriol supplementation in increasing the serum calcium content may be in part explained by lack of calcium supplementation and possible counter absorptive effect of calcitriol of bone metabolism theorized here.

Our data was consistent with the inhibiting effect of calcitriol on bone osteoblastic activity. This was accompanied by lower values for serum calcium in the calcitriol supplemented group, which may point to the simultaneous inhibition of osteoclastic activity in this group. Considering that bone osteoclastic activity that leads to release of calcium from bone tissue is the underlying cause for space flight induced osteoporosis [17] herein it is hypothesized that calcitriol suppresses bone turnover thereby preventing osteoporosis in rat tail suspension model. The inhibition of bone turnover by calcitriol is reflected in lower values for OC, ALP and serum calcium content in our study though they were not statistically significant pointing to the need for further studies in future.

We note the following limitations to our study. First, limited number of rats was included in our study which may result in low power of the study. Although the sample size calculation and power assessments demonstrated that our study has the 90% power to detect the significant difference the study results did not become significant.

Second, change in ALP and OC levels obey a bimodal pattern during tail suspension. This pattern was reported by a study conducted on different strains of mice indicating that although serum levels of ALP and OC increase after 2 weeks of suspension, their values tend to restore in the next week [18]. This pattern point to the need for multiple sampling during tail suspension studies to detect significant differences.

Third, we did not assess other biomarkers of bone metabolism. This could limit our interpretation of cellular responses to treatment regimens. However objectively, it is clear that biomarkers of bone formation that we studied here, account for osteoblastic activity in the process of bone turn over and their results accompanied by the measurement of other markers of bone metabolism like vitamin D and calcium would yield to appropriate assessment of bone metabolism in the study.

Future studies with larger study populations and measurement of other biomarkers of bone metabolism are recommended to support the result of our study.

## Conclusion

We have studied the biomarkers of bone formation to assess the effect of calcitriol supplementation on bone cellular responses during weightlessness in rats by means of tail suspension model that is the model of choice for space flight simulation in laboratory.

In conclusion, supplementation of daily diet with activated vitamin D could result in lower levels of OC and ALP, along with lower levels of serum calcium in hindlimb suspended rats compared to tail suspension alone in animal model of weightlessness. This was in favor of inhibiting effect of calcitriol on bone turnover and thereby inhibiting the process of osteoporosis in tail suspended rats. Our study possess some limitations including low number of study groups leading to statistically not significant results pointing to the need for future studies to test the hypothesized role for calcitriol supplementation on bone metabolism in space flight animal models.
